# Supplementary material for: A Label-Free Electrical Impedance Spectroscopy for Detection of Clusters of Extracellular Vesicles Based on Their Unique Dielectric Properties
Source: Biosensors (Basel). 2022 Feb 9;12(2):104. doi: 10.3390/bios12020104 (PMC8869858; doi:10.3390/bios12020104)
Supplement: Supplementary file 1 [file biosensors-12-00104-s001.zip › biosensors-1563651-supplementary.pdf]

biosensors

Article

A Label-Free Electrical Impedance Spectroscopy for Detection of Clusters of Extracellular Vesicles Based on Their Unique Dielectric Properties

Yuqian Zhang [1,2], Kazutoshi Murakami [3], Vishnupriya J. Borra [3], Mehmet Ozgun Ozen [4,5], Utkan Demirci [4,5], Takahisa Nakamura [3,6,7] and Leyla Esfandiari [8,9,10,*]

1. Methods

Preparation of liposomes with different membrane composition. Liposomes with different membrane composition were synthesized by systematically changing the molar ratio of cholesterol (CH) and L-α-Phosphatidylcholine (PC):CH/PC = 1:10 and CH/PC = 10:1. Liposomes were prepared by thin-film hydration method followed by extrusion [1]. 66.8 μL of 10 mg/mL CH in chloroform and 133.2 μL of 100 mg/mL PC in chloroform were mixed in a round bottom flask to prepare 200 μL of CH/PC liposome (1:10 molar ratio); 196 μL of 10 mg/ml CH in chloroform and 4 μL of 100 mg/mL PC in chloroform were mixed to prepare 200 μL of CH/PC liposomes (10:1 molar ratio). The chloroform in the mixtures was evaporated in a rotary evaporator at 40 °C water bath overnight, forming the thin lipid film. The dry lipid film was hydrated with 1 mL PBS buffer while vortexing and sonicating to form liposome suspension. The milky crude liposome suspension was frozen in dry ice for 5 min followed by thawing for 15 min at room temperature and the freeze-thaw cycle was repeated 10 times. The liposomes were extruded for 11 passes through two stacked polycarbonate filters with the pore sizes of 1 μm assembled into the extruder (Mini extruder, Avanti Polar Lipids). The extrusion procedure was repeated 3 times using 400 nm, 200 nm and 100 nm polycarbonate membranes sequentially to form liposomes of 100 nm in diameter. Upon the extrusion, size and the concentration of the liposomes were measured using the Nanoparticle Tracking Analysis (Nanosight NS3000, Malvern Panalytical). In the case of $CH:PC_{(1:10)}$, the mean diameter was measured as 101.6 ± 28.6 nm at the concentration of 1.7×10^{13} per mL; and in the case of $CH:PC_{(10:1)}$, the mean diameter was measured as 105.9 ± 30.1 nm at the concentration of 4.5×10^{12} per mL (Figure S1).

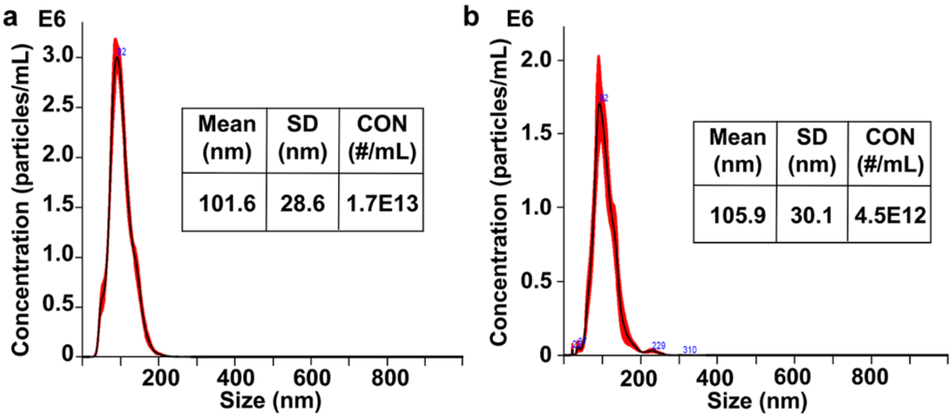

Figure S1. (**a**) NTA results of liposomes $CH:PC_{(1:10)}$. (**b**) NTA result of liposomes $CH:PC_{(10:1)}$ synthesized by extrusion method.

Preparation of EVs from mouse primary hepatocytes. Green fluorescent proteins (GFP) were tagged with an EV target sequence (EXtS) followed by breeding to an albumin-

cre mice. By utilizing the Lox66 and Lox71 sequences, the EXtS tagged GFP gene (EXtS-GFP) is "flipped" on when Cre-recombinase is expressed [2] which, in turn, leads to expression of the EXtS-GFP (Figure S2a). Primary hepatocytes were isolated from EXtS-GFP (Alb-Cre negative) and EXtS-GFPAlb-cre (Alb-Cre positive) mice by two step perfusion method [3]. 1 × 10^7 cells were cultured in 150 mm culture dishes in DMEM containing 10% fetal bovine serum and 1% Anti-Anti. Once hepatocytes were adhered to the plate, these cells were cultured in DMEM containing 10% EV-depleted fetal bovine serum and 1% Anti-Anti for 24 h before collecting the conditioned media for EVs isolation. Figure S2b showed the expression of fluorescent EXtS-GFP in the primary hepatocytes of the albumin-Cre mice. Western blot analysis of EVs collected from culture media of primary hepatocytes was presented in Figure S2c, and it confirmed that EVs were labeled with GFP when Alb-Cre expressed [4]. Western blot analysis on lysates as a control, demonstrated the expression of the fusion gene (GFP) in the hepatocyte.

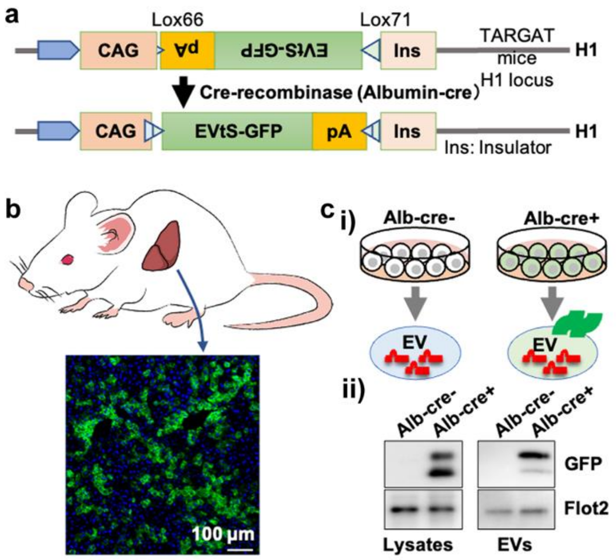

Figure S2. (**a**) An in vivo strategy to label EVs with GFP in a hepatocyte-specific manner. Recombination results in an inversion, and the region between the lox66-lox71 sites is reversed. EVtS-GFP can be expressed under the control of Cre-recombinase. (**b**) EXtS-GFP line was breeding with albumin-cre mice (EXtS-GFPAlb-cre mice); Microscopy image showed the representative patterns of GFP expression in liver cells; (**c**) (i) Extracellular vesicles were collected from the conditioned medium of primary hepatocytes from EVtS-GFP mice (Alb-Cre negative and positive); (ii) Western blot analysis of GFP in collected EVs and lysates. The Flotillin-2/Flot2 was used as an EV marker.

Size and concentration of extracted EVs were measured using NTA. In the case of EVs from control sample (GFP-), the mean diameter was measured as 127.1 ± 80.8 nm with the concentration of 8.27 × 10^9 per mL; in the case of EVs with GFP in the culture medium (GFP+), the mean diameter was measured as 111.6 ± 49.9 nm with the concentration of 9.39 × 10^9 per mL (Figure S3).

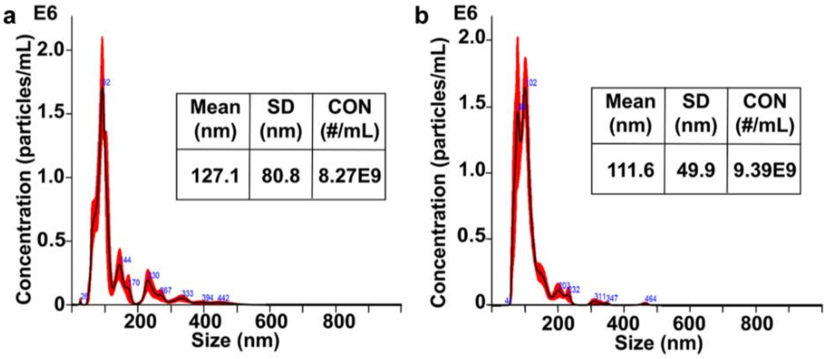

Figure S3. NTA results of EVs extracted from culture media of (**a**) control (**b**) green fluorescent protein (GFP+) transgenic mouse primary hepatocytes.

Preparation of EVs from human hepatocellular carcinoma (HuH-7). HuH-7 cells (6 × 10^6) were seeded in 150 mm culture dishes and maintained in DMEM media supplemented with 10% (v/v) EV-depleted FBS and Anti-Anti in 5% CO_2 at 37 °C. After 24 h, cells were washed with warmed PBS once and DMEM media supplemented with 10% (v/v) EV-depleted FBS, anti-anti and DMSO (control), 75 μM sodium palmitate acid (PA), and 75 μM sodium palmitate acid plus 10 μM GW4869 (PA + GW) was replaced separately (pre-treatment). After 4 h, cells were washed again with warmed PBS twice and the same DMEM media as pre-stimulation was replaced (treatment). After 20 h, the culture supernatant was collected. Subsequently, the procedure of MagCapture Exosome Isolation Kit PS was followed for extraction of EVs from cell culture. Isolated EVs were aliquoted and kept at −80 °C until use. In the case of EVs from control sample, the mean diameter was measured as 115.5 ± 46.4 nm with the concentration of 6.55 × 10^{10} per mL; in the case of EVs in palmitate acid (PA), the mean diameter was measured as 132.8 ± 68.2 nm with the concentration of 7.84 × 10^{10} per mL; in the case of EVs in the mixture of palmitate and GW4869, the mean diameter was measured as 120.9 ± 62.2 nm with the concentration of 6.59 × 10^{10} per mL (Figure S4).

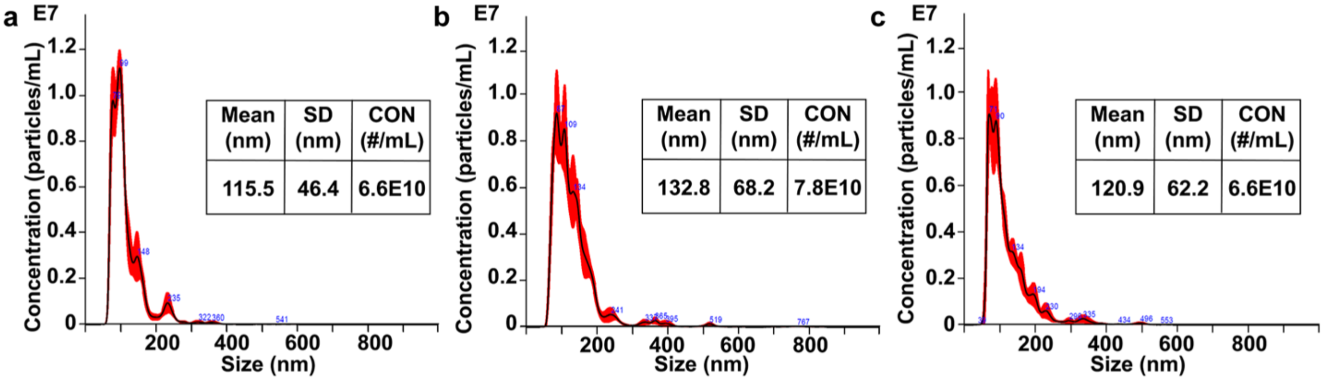

Figure S4. NTA results of EVs extracted from culture medium of HuH-7 cell lines (**a**) control (**b**) palmitate acid (**c**) mixture of palmitate acid and GW4869.

Preparation of EVs from HUVEC and MDA-MB-231 cell lines. MDA-MB-231 cells were cultured in Dulbecco's modified Eagle medium (DMEM) supplemented with 10% fetal bovine serum (FBS) and 1% penicillin-streptomycin in T75 flasks with 5% CO_2 at 37 °C till the culture is confluent. HUVEC cell line was cultured with endothelial cell culture media supplemented with 2% fetal calf serum (FCS), 0.4% endothelial cell growth supplement, 0.1 ng/mL recombinant human epidermal growth factor, 1 ng/mL recombinant human basic fibroblast growth factor, 90 μg/mL heparin, 1 μg/mL hydrocortisone, and 1% penicillin-streptomycin in T75 flasks with 5% CO_2 at 37 °C till the culture is confluent. Then, after removing the culture media from the flasks, the cultures were washed with 15

mL PBS (5X) to eliminate remaining FBS/FCS. For the EV production phase, cells were cultured with fresh media supplemented with 5% exosomes-depleted FBS instead of FBS and FCS keeping other supplements constant for 48 h. After two days, EV production media were withdrawn from flasks, and centrifuged at 4 °C for 20 min at 2000 g to separate cell debris and cells. The centrifuged supernatant was filtered with 0.22 µm to remove bigger contaminating particles. The prepared supernatant was stored at −80 °C if it was not used at the time of preparation. The fresh/thawed supernatant was used to isolate EVs using a novel, in house developed, size-based EV isolation tool, Exosome Total Isolation Chip (ExoTIC). The working principle and details of the platform were reported earlier [5]. Briefly, we employed four serially connected chips prepared using filters with different pore-sizes (30 nm, 50 nm, 80 nm, and 100 nm) to sort EVs according to their size.

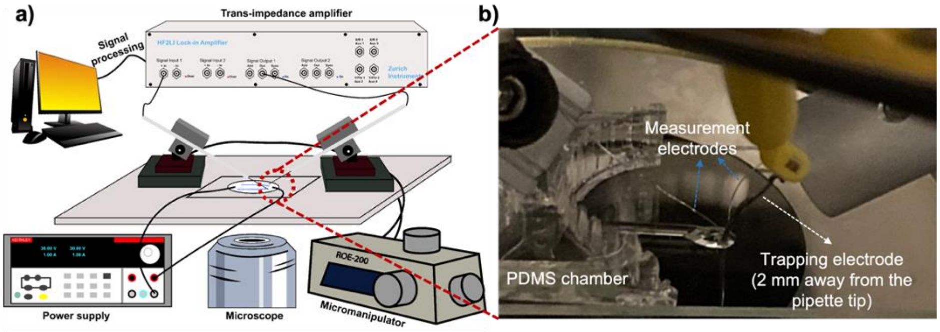

Figure S5. (**a**) Diagram of the electrical impedance measurement system (not to scale). The system consists of two modules: (1) a set of platinum electrodes (OD = 0.51 mm) placed across the micropipette (length = 3 cm), to apply 10 V/cm DC for vesicles entrapment; (2) another set of platinum electrodes (OD = 130 µm) were preciously placed across the pipette tip 20 µm apart *via* a multi-micromanipulator system, and the impedance signals were recorded and analyzed with a trans-impedance amplifier. (**b**) A picture of the setup on the microscope stage showing a micropipette tip immersed in 10 µL PBS solution. Polydimethylsiloxane (PDMS) chamber with a 1 mm diameter opening was fabricated to fix the position of the micropipette. Trapping electrode was placed 2 mm away from the pipette tip.

1.1. Impedance data analysis

After trapping a cluster of particles (e.g. liposomes) at the micropipettes' tip, the measurement was conducted by logarithmically sweeping 500 points at a wide frequency spectrum (0.5 MHz to 50 MHz), and the signals were recorded as amplitude (V) and phase (°) as depicted in Figure S6a. The impedance (Z_{mix} Figure S6b) was further calculated based on Eq. S1 derived from the equivalent circuit (Figure S7), in which V_{out} represents the recorded output signal (amplitude (V) and phase (°)). R_{in} (50 Ω) and R_s (50 Ω) are intrinsic resistors coupled in the impedance analyzer, Vin is the input voltage (100 mV).

$$V_{out} = V_{in} \times \frac{R_{in}}{Z_{mix} + R_s} \tag{S1}$$

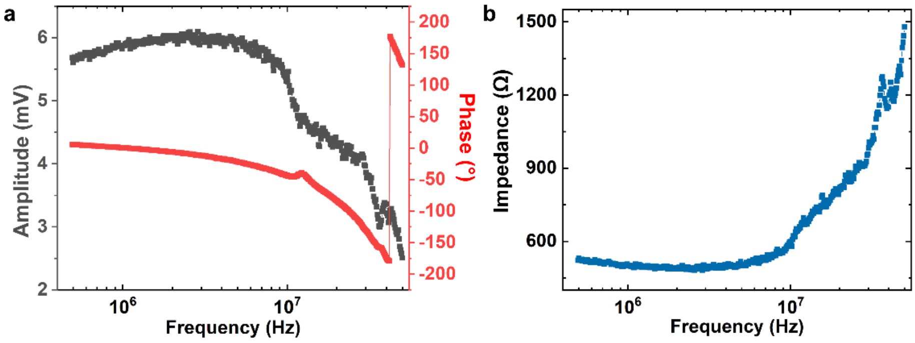

Figure S6. Data analysis based on the raw data obtained from the HF2LI impedance analyzer. (**a**) The output signals (raw data) was record as amplitude (mV) and Phase (°) at a wide frequency spectrum (0.5 MHz to 50 MHz). (**b**) The impedance spectrum obtained by converting the output amplitude signal to impedance based on the equivalent circuit.

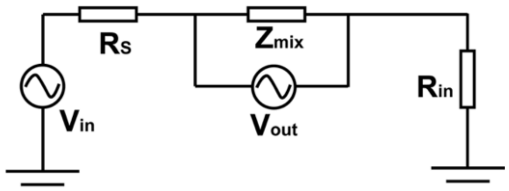

Figure S7. Schematic diagram of the HF2LI impedance analyzer. R_{in} (50 Ω) and R_s (50 Ω) are intrinsic resistors coupled in the impedance analyzer. Z_{mix} represents the impedance of a cluster of particles cluster, and V_{in} is the input voltage (100 mV) and V_{out} represents the measured output signal.

1.2. Magnitude Opacity

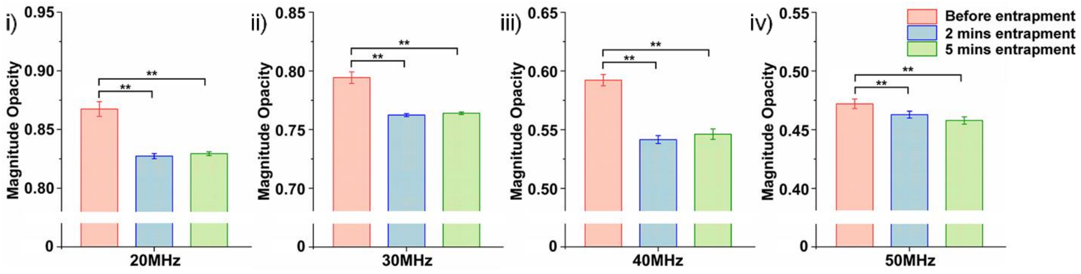

Figure S8. Experimental data showing the magnitude opacity comparison among empty pipette (before entrapment) and liposome clusters extracted at two different time intervals at 20MHz–50MHz. $^{**}P < 0.05$.

2. Mathematical Model for Particles in Solution

The equivalent circuit was modified from the Foster and Schwan circuit model that has been established in the cell cytometry [6]. The cell in this model is represented as a capacitor (C_P) for its' membrane capacitance and a resistor (R_P) to define its' cytoplasm conductivity [7,8]. Similar equivalent circuit (Figure S9a) was utilized here to model the cluster of liposomes in the solution represented by (Z_{mix}) [9,10]. The particles' impedance (Z_{mix}) is connected in series with an electrical double-layer capacitance (C_{dl}) and in parallel with a stray capacitance (C_{stray}). Also, a lead inductance (L_{ld}) and a resistance (R_{ld}) represent the connecting cables of the impedance analyzer that were included in the circuit [11,12]. The constructed equivalent circuit provided a more straightforward impedance response to the alternations of particles' intrinsic capacitance and conductance. Based on the circuit model, the variation on particles' dielectric properties lead to changes (ΔCp and ΔR_P) on their effective C_P and R_P, and consequently a shift in their magnitude opacity, as

illustrated in (Figure S9b). The decrease of the particles' capacitance (C_P–ΔC_P) or the increase of the particles' resistance (R_P–ΔR_P) would result in the elevated magnitude opacity, and vice versa.

The impedance of particles in suspension (Z_{mix}) was estimated based on Maxwell's mixture theory (Figure S9c) [8,13–15], which was related to the complex permittivity of the particles ($\tilde{\varepsilon}_{mix}$) through Equations S2 and S3:

$$Z_{mix} = \frac{1}{j\omega\tilde{\varepsilon}_{mix}G_f} \quad \text{(S2)}$$

$$\tilde{\varepsilon}_{mix} = \tilde{\varepsilon}_m \frac{1+2\phi\tilde{f}_{CM}}{1-\phi\tilde{f}_{CM}} \quad \text{with} \quad \tilde{f}_{CM} = \frac{\tilde{\varepsilon}_p-\tilde{\varepsilon}_m}{\tilde{\varepsilon}_p+2\tilde{\varepsilon}_m} \quad \text{(S3)}$$

in which G_f represents the geometrical constant, representing the ratio of electrodes surface area to the electrodes gap A/g (m) for an ideal parallel plates electrode system. The complex permittivity $\tilde{\varepsilon}_{mix}$ of the particles is given in Equation (S3) [8,16], where $\tilde{\varepsilon} = \varepsilon - j\frac{\sigma}{\omega}$ is the complex permittivity, ω is the angular frequency, ε and σ are permittivity and conductivity. $\tilde{\varepsilon}_p$ and $\tilde{\varepsilon}_m$ refer to the complex permittivity of the particles and suspending medium, respectively. $\tilde{f}_{CM}$ is the Clausius-Mossotti factor which describes the frequency-dependent polarizability of particles in solution, and ϕ represents the volume fraction of particles in suspending medium.

Liposomes in suspension were modeled as a single-shell model [14,15] due to their similar structure of a lipophilic shell and an aqueous core. The complex permittivity of the particle is a function of the dielectric properties of its membrane and cytosol, which is described in Equation (S4).

$$\tilde{\varepsilon}_p = \tilde{\varepsilon}_{mem} \frac{\gamma^3+2\left(\frac{\tilde{\varepsilon}_i-\tilde{\varepsilon}_{mem}}{\tilde{\varepsilon}_i+2\tilde{\varepsilon}_{mem}}\right)}{\gamma^3-\left(\frac{\tilde{\varepsilon}_i-\tilde{\varepsilon}_{mem}}{\tilde{\varepsilon}_i+2\tilde{\varepsilon}_{mem}}\right)} \quad \text{with} \quad \gamma = \frac{R+d}{R} \quad \text{(S4)}$$

in which the $\tilde{\varepsilon}_{mem}$ and $\tilde{\varepsilon}_i$ represent the complex permittivity of the membrane and cytosol. The complex permittivity is given by $\tilde{\varepsilon} = \varepsilon - j\frac{\sigma}{\omega}$, where ε is the permittivity and σ is the conductivity. The permittivity and the conductivity of the liposome membrane were estimated with equations: $\varepsilon_{mem}(F/m) = C_{mem}(F/m^2) \times d(m)$ and $\sigma_{mem}(S/m) = d(m)/R_{mem}(\Omega \cdot m^2)$, in which d represents the thickness of the membrane (5 nm). The capacitance and resistance of the liposomes were estimated based on the lipid molar ratio. The relative permittivity of $CH{:}PC_{(1:10)}$ and $CH{:}PC_{(10:1)}$ were approximated as 2.26 and 3.33, respectively. The conductivity of $CH{:}PC_{(1:10)}$ and $CH{:}PC_{(10:1)}$ were approximated as 3.16×10^{-9} S/m and 3.37×10^{-10} S/m, respectively. The relative permittivity and conductivity of internal solution of liposomes are 80 and 1.6 S/m.

COOH-PS beads are considered as solid dielectric spheres, which their permittivity and conductivity are frequency-independent [17]. The complex permittivity of the bead was calculated using $\tilde{\varepsilon_p} = \varepsilon_p - j\frac{\sigma_p}{\omega}$, with the permittivity ε_p of $2.55\varepsilon_0$ [18]. The conductivity of the particle is defined as $\sigma_p = \sigma_{bulk} + \frac{2K_s}{r}$. Where σ_{bulk} represents the conductivity of bulk material, and it is usually taken as 0 due to the inherently low conductivity of polystyrene material [18,19]. K_s is the surface conductance and r is the radius of the bead. Thus, the conductivity of the COOH-PS bead is highly dependent on the surface charge and the size. However, it is difficult to accurately estimate the surface conductance of the COOH-PS beads without knowing their crossover frequency and thus, we used the $\frac{K_s}{r}$ = 0.25 nS/μm, which was reported by Morgan's group [20].

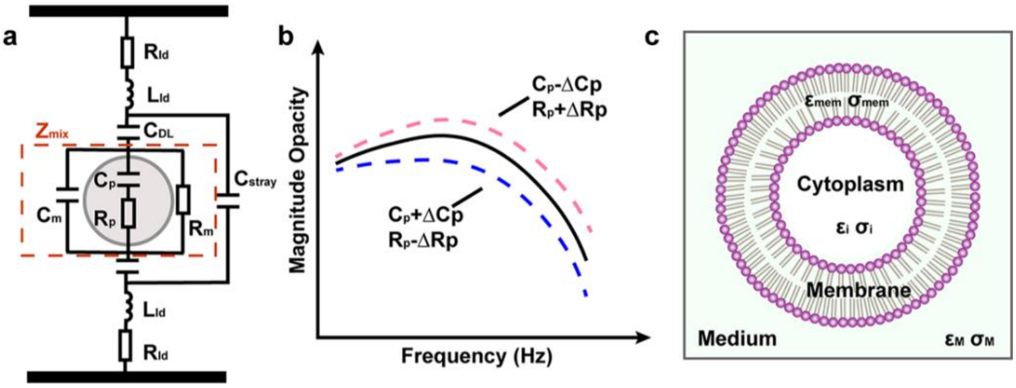

Figure S9. (**a**) An equivalent circuit model for the impedance measurement system. Liposomes in suspension are modeled as a capacitor C_P (membrane) and a resistor R_P (cytoplasm) in series based on the Foster and Schwan's simplified circuit model; (**b**) A magnitude opacity spectrum that exemplifies impedance shifts (dash lines) upon a resistance change (ΔR_P) and capacitance change (ΔC_P) of particles; (**c**) Diagram of a single-shell model, representing a single vesicle in suspension. ε_m and σ_m represent the permittivity and conductivity of the medium; ε_{mem} and σ_{mem} depict the permittivity and conductivity of the membrane; ε_i and σ_i describe the permittivity and conductivity of the lumen.

The impedance of the system is calculated by incorporating the Z_{mix} in to the equivalent circuit as illustrated in Figure S9a. C_{dl} presented in the electrode-electrolyte interface which caused by electrode polarization effect and the C_{stray} existed between two proximal electrodes at different potentials, which induced by storage of opposite electric charges on the electrodes [21]. C_{stray} and C_{dl} were estimated by fitting the measured impedance spectra of electrolyte solutions with known conductivities, into the Constant Phase Element (CPE) circuit component [6,22,23]. The value of the lead inductance (L_{ld}) and resistance (R_{ld}) were obtained by measuring the impedance of the analyzer cables at open circuit, short circuit, and 1 kΩ resistor load [11,12]. Computation of the mathematical model was performed in MATLAB with the following parameters: C_{dl} = 15 μF, C_{stray} = 0.8 pF, L_{ld} = 0.57 μH and R_{ld} = 3.7 μΩ, volume fraction ϕ = 0.1 and geometric constant G_f = 7×10^{-3}. In order to keep consistent with the parameter used in the experiments, the estimated impedance was converted to amplitude (V_{out}) based on the impedance analyzer circuit model in Figure S7. The output amplitude is calculated using Equation (S5):

$$V_{out} = V_{in} \times \frac{R_{in}}{Z_{mix} + R_s} \quad \text{(S5)}$$

where V_{in} is the input voltage 0.1 V, Rs = 50 Ω and Rin = 50 Ω. The magnitude opacity was calculated by dividing V_{out} measured at all frequencies by V_{out} at 0.5 MHz.

3. Detection of EVs With Different Membrane Compositions

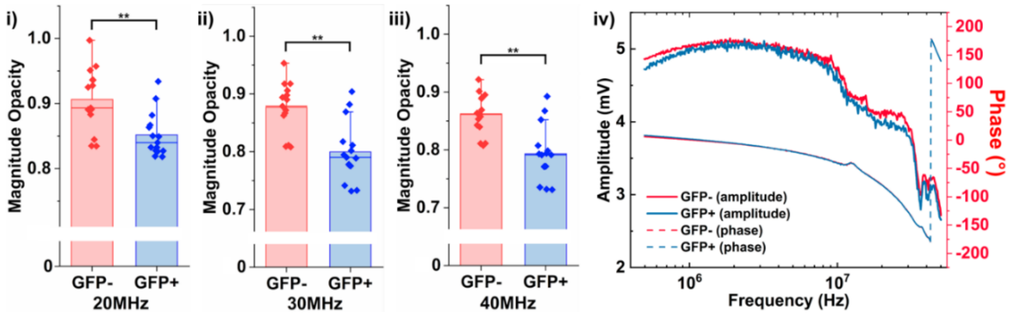

Figure S10. The magnitude opacity comparison of EVs derived from wild type primary hepatocytes (GFP−) and GPF+ hepatocytes at (**i**)–(**iii**) 20 MHz–40 MHz. (***P* < 0.05, n = 15); (**iv**) Bode plot of GFP− and GFP+ EVs as a function of frequency measured between 0.5 MHz to 50 MHz.

4. Detection of EVs Secreted from Cells Treated Under Different Culture Conditions

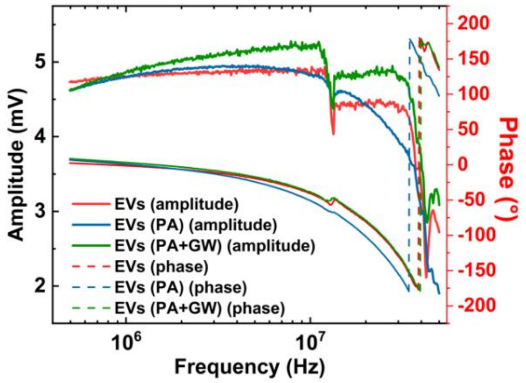

Figure S11. Bode plot (amplitude (mV) and phase (°)) of EVs from human hepatocellular carcinoma cell lines under: normal culture medium (EVs), PA treated condition (EVs (PA)) and the mixture of PA and GW4869 (EVs (PA + GW)) treated conditions, as a function of frequency measured between 0.5 MHz to 50 MHz.

5. Differentiating EVs from Lipoproteins

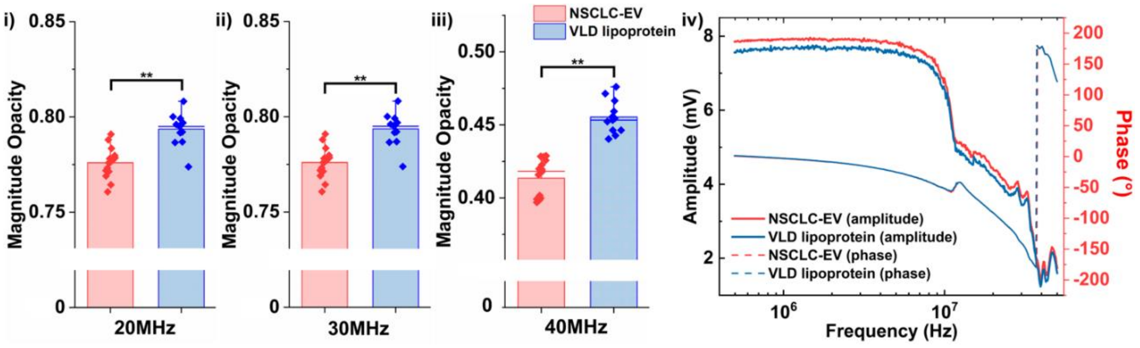

Figure S12. Magnitude opacity comparison of EVs derived from A549 non-small cell lung cancer (NSCLC) cell line and very low-density (VLD) lipoprotein at (**i**)–(**iii**) 20 MHz–40 MHz. (** $P < 0.05$, n = 12); (**iv**) Bode plot of NSCLC-EV and VLD lipoprotein as a function of frequency measured between 0.5 MHz to 50 MHz.

6. Detection of EVs Derived from Different Cellular Origins

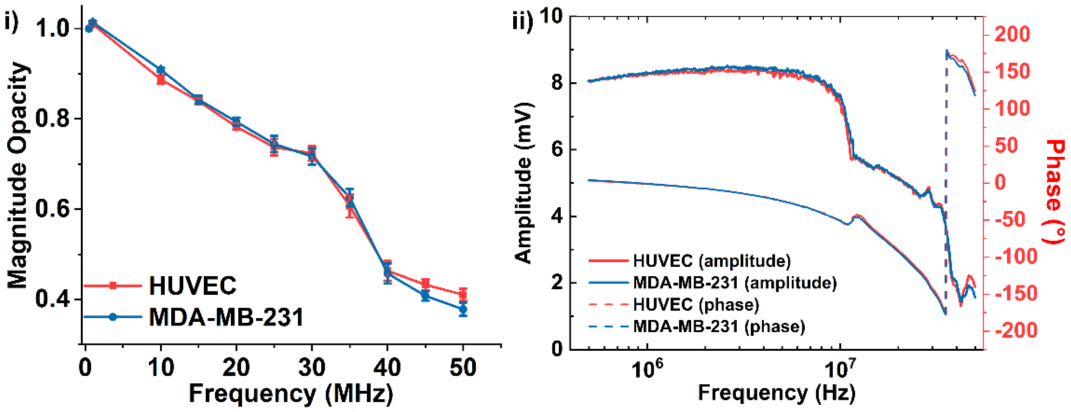

Figure S13. (i) Magnitude opacity spectrum of EVs extracted from human umbilical vein endothelial cells (HUVEC) and epithelial human breast cancer (MDA-MB-231) cells; (**ii)** Bode plot of EVs from HUVEC and MDA-MB-231 cells as a function of frequency measured between 0.5 MHz to 50 MHz.

7. Detection of EVs of Different Size Distribution

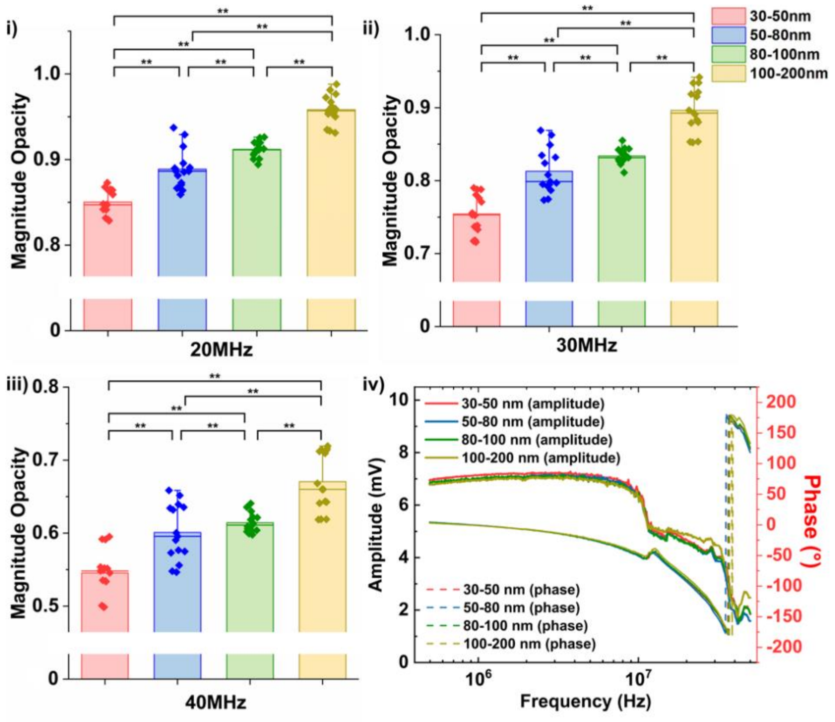

Figure S14. The magnitude opacity of EVs derived from MDA-MB-231 cell line with different size range measured at (**i**)–(**iii**) 20 MHz–40 MHz. (**P < 0.05, n = 15); (**iv**) Bode plot of four EVs subsets as a function of frequency measured between 0.5 MHz to 50 MHz.

References

1. Zhang, H. Thin-Film Hydration Followed by Extrusion Method for Liposome Preparation. In *Liposomes: Methods and Protocols*, D'Souza, G.G.M., Ed.; Springer New York: New York, NY, 2017; pp. 17-22.
2. Zhang, Z.; Lutz, B. Cre recombinase-mediated inversion using lox66 and lox71: method to introduce conditional point mutations into the CREB-binding protein. *Nucleic Acids Res* 2002, *30*, e90, doi: 10.1093/nar/gnf089
3. Salem, E.S.B.; Murakami, K.; Takahashi, T.; Bernhard, E.; Borra, V.; Bethi, M.; Nakamura, T. Isolation of Primary Mouse Hepatocytes for Nascent Protein Synthesis Analysis by Non-radioactive L-azidohomoalanine Labeling Method. *J Vis Exp* 2018, doi:10.3791/58323.
4. Shen, B.; Wu, N.; Yang, J.M.; Gould, S.J. Protein targeting to exosomes/microvesicles by plasma membrane anchors. *J. Biol. Chem.* 2011, *286*, 14383-14395, doi:10.1074/jbc.M110.208660.
5. Liu, F.; Vermesh, O.; Mani, V.; Ge, T.J.; Madsen, S.J.; Sabour, A.; Hsu, E.-C.; Gowrishankar, G.; Kanada, M.; Jokerst, J.V.; et al. The Exosome Total Isolation Chip. *ACS Nano* 2017, *11*, 10712-10723, doi:10.1021/acsnano.7b04878.
6. Schwan, H.P. ELECTRODE POLARIZATION IMPEDANCE AND MEASUREMENTS IN BIOLOGICAL MATERIALS*. *Ann. N. Y. Acad. Sci.* 1968, *148*, 191-209, doi:https://doi.org/10.1111/j.1749-6632.1968.tb20349.x.
7. Foster, K.R.; Schwan, H.P. Dielectric properties of tissues and biological materials: a critical review. *Crit. Rev. Biomed. Eng.* 1989, *17*, 25-104.
8. Sun, T.; Morgan, H. Single-cell microfluidic impedance cytometry: a review. *Microfluid. Nanofluidics.* 2010, *8*, 423-443, doi: https://doi.org/10.1007/s10404-010-0580-9
9. Ren, J.; He, W.; Zheng, L.; Duan, H. From structures to functions: insights into exosomes as promising drug delivery vehicles. *Biomater. Sci.* 2016, *4*, 910-921, doi:10.1039/C5BM00583C.
10. Théry, C.; Zitvogel, L.; Amigorena, S. Exosomes: composition, biogenesis and function. *Nat. Rev. Immunol,* 2002, *2*, 569-579, doi: https://doi.org/10.1038/nri855
11. Sabuncu, A.C.; Zhuang, J.; Kolb, J.F.; Beskok, A. Microfluidic impedance spectroscopy as a tool for quantitative biology and biotechnology. *Biomicrofluidics* 2012, *6*, 034103, doi:10.1063/1.4737121.
12. Raicu, V. A simple theoretical and practical approach to measuring dielectric properties with an open-ended coaxial probe. *Meas. Sci. Technol.* 1995, *6*, 410-414, doi:10.1088/0957-0233/6/4/011.
13. Asami, K. Dielectric dispersion in biological cells of complex geometry simulated by the three-dimensional finite difference method. *J. Phys. D: Appl. Phys.* 2006, *39*, 492-499, doi:10.1088/0022-3727/39/3/012.
14. Sun, T.; Gawad, S.; Green, N.G.; Morgan, H. Dielectric spectroscopy of single cells: time domain analysis using Maxwell's mixture equation. *J. Phys. D: Appl. Phys.* 2006, *40*, 1-8, doi:10.1088/0022-3727/40/1/s01.

15. Maxwell, J.C. *A treatise on electricity and magnetism*; Clarendon press: 1873; Volume 1.
16. Holmes, D.; Pettigrew, D.; Reccius, C.H.; Gwyer, J.D.; van Berkel, C.; Holloway, J.; Davies, D.E.; Morgan, H. Leukocyte analysis and differentiation using high speed microfluidic single cell impedance cytometry. *Lab. Chip.* 2009, *9*, 2881-2889, doi:10.1039/B910053A.
17. Sun, T.; Bernabini, C.; Morgan, H. Single-Colloidal Particle Impedance Spectroscopy: Complete Equivalent Circuit Analysis of Polyelectrolyte Microcapsules. *Langmuir* 2010, *26*, 3821-3828, doi:10.1021/la903609u.
18. Sun, T.; Holmes, D.; Gawad, S.; Green, N.G.; Morgan, H. High speed multi-frequency impedance analysis of single particles in a microfluidic cytometer using maximum length sequences. *Lab. Chip.* 2007, *7*, 1034-1040, doi:10.1039/B703546B.
19. Vahey, M.D.; Voldman, J. High-throughput cell and particle characterization using isodielectric separation. *Anal. Chem.* 2009, *81*, 2446-2455, doi:10.1021/ac8019575.
20. Cui, L.; Holmes, D.; Morgan, H. The dielectrophoretic levitation and separation of latex beads in microchips. *Electrophoresis* 2001, *22*, 3893-3901, doi:10.1002/1522-2683(200110)22:18<3893::AID-ELPS3893>3.0.CO;2-2.
21. Zhang, G.; Zhu, R. Effect of Parasitic Capacitance on Impedance Measurement and Model Extraction. *Electroanalysis* 2010, *22*, 351-358, doi:10.1002/elan.200900324.
22. Yoon, G. Dielectric properties of glucose in bulk aqueous solutions: Influence of electrode polarization and modeling. *Biosens. Bioelectron.* 2011, *26*, 2347-2353, doi:https://doi.org/10.1016/j.bios.2010.10.009.
23. Kaatze, U. Complex permittivity of water as a function of frequency and temperature. *J. Chem. Eng. Data* 1989, *34*, 371-374, doi:10.1021/je00058a001.
